# Cost-effectiveness analysis of ranibizumab for retinal vein occlusion patients in China from the societal perspective

**DOI:** 10.1186/s12886-021-01997-1

**Published:** 2021-05-24

**Authors:** Weiyi Ni, Jia Liu, Yawen Jiang, Jing Wu

**Affiliations:** 1 Department of Pharmaceutical and Health Economics, University of Southern California, Los Angeles, CA USA; 2 School of Pharmaceutical Science and Technology, Tianjin University, Nankai District, Room 209, 24th building, 92th Weijin Road, Tianjin, 300072 China; 3 Center for Social Science Survey and Data, Tianjin University, Tianjin, China; 4 School of Public Health (Shenzhen), Sun Yat-Sen University, Shenzhen, Guangdong China

**Keywords:** Ranibizumab, BRVO, CRVO, Cost-effectiveness

## Abstract

**Background:**

Clinical trials in China have demonstrated that ranibizumab can improve the clinical outcomes of branch retinal vein occlusion (BRVO) and central retinal vein occlusion (CRVO). However, no economic evaluation of ranibizumab has been conducted among Chinese patient population.

**Methods:**

To provide insights into the economic profile of ranibizumab among Chinese RVO population, a Markov state-transition model was used to predict the outcomes of ranibizumab comparing to laser photocoagulation and observational-only care from the societal perspective. This model simulated changes in patient visuality, quality-adjusted of life years (QALY), medical costs, and direct non-medical costs of individuals with visual impairment due to BRVO or CRVO in lifetime. The base-case analysis used an annual discount rate of 5% for costs and benefits following the China Guidelines for Pharmacoeconomic Evaluations. Deterministic and probabilistic sensitivity analyses were performed to test the robustness of the model.

**Results:**

The base-case incremental cost-effectiveness ratio (ICER) comparing ranibizumab to laser photocoagulation was ¥65,008/QALY among BRVO patients and was ¥65,815/QALY among CRVO patients, respectively. Comparing to the 2019 gross domestic product (GDP) per capita of ¥71,000, both two ICERs were far below the cost-effective threshold at three times of GDP per capita (¥213,000). The deterministic and probabilistic sensitivity analyses demonstrated the base-case results were robust in most of the simulation scenarios.

**Conclusion:**

The current Markov model demonstrated that ranibizumab may be cost-effective compared with laser photocoagulation to treat BRVO and cost-effective compared to observation-only care to treat CRVO in China from the societal perspective.

**Supplementary Information:**

The online version contains supplementary material available at 10.1186/s12886-021-01997-1.

## Background

Retinal vein occlusion (RVO), including the central retinal vein occlusion (CRVO) and branch retinal vein occlusion (BRVO), is a prevalent vision-threatening disease. The International Eye Disease Consortium reported that the prevalence of RVO in the USA, Europe, Asia, and Australia was 0.52% for any RVO, 0.44% for BRVO, and 0.08% for CRVO [1].

Macular edema (ME) is a common visual complication and a primary cause of visual loss in patients with RVOs. ME secondary to BRVO is generally treated with laser photocoagulation or anti-vascular endothelial growth factor (VEGF) agents. Clinical trials have shown that anti-VEGF therapies are more effective at improving best-corrected visual acuity (BCVA) than laser photocoagulation [2]. More, although anti-VEGF therapy has better clinical efficacy, observation-only care is still a common treatment for CRVO, especially among newly diagnosed patients [3–5].

Laser photocoagulation used to be the standard care for ME [3, 6]. However, in cases of severe ME, retinal swelling may reduce the penetration power of laser, leading to poor treatment effects, which is a major drawback of laser photocoagulation. Although anti-VEGF agents are shown to be more beneficial in treating RVO patients through clinical trials, the treatment itself requires multiple repeat intravitreal injections to maintain the clinical outcomes. Consequently, it is likely to incur higher medication expenditure of the healthcare system.

Recently, BLOSSOM and CAMELLIA clinical trials in China have demonstrated that ranibizumab is more effective than observation-only care for BRVO and CRVO [7, 8]. Hence, the introduction of ranibizumab into the Chinese healthcare system may represent an opportunity to improve the health outcomes of RVO patients in China. However, its incremental clinical effectiveness still has to be weighed against higher acquisition expenditure, including the cost of ranibizumab and administration charge. Although studies in Europe demonstrated that ranibizumab is cost-effective comparing to laser photocoagulation and observation-only care, the conclusion may not be portable to the Chinese healthcare system because costs of medical services, medical resource utilization and productivity loss can vary substantially across the two regions. To provide insights into the economic profile of ranibizumab among Chinese RVO population, the current study used a Markov decision model to predict the outcomes of ranibizumab comparing to laser photocoagulation and observational-only care from the societal perspective.

## Methods

### Model structure

**Fig. 1:**
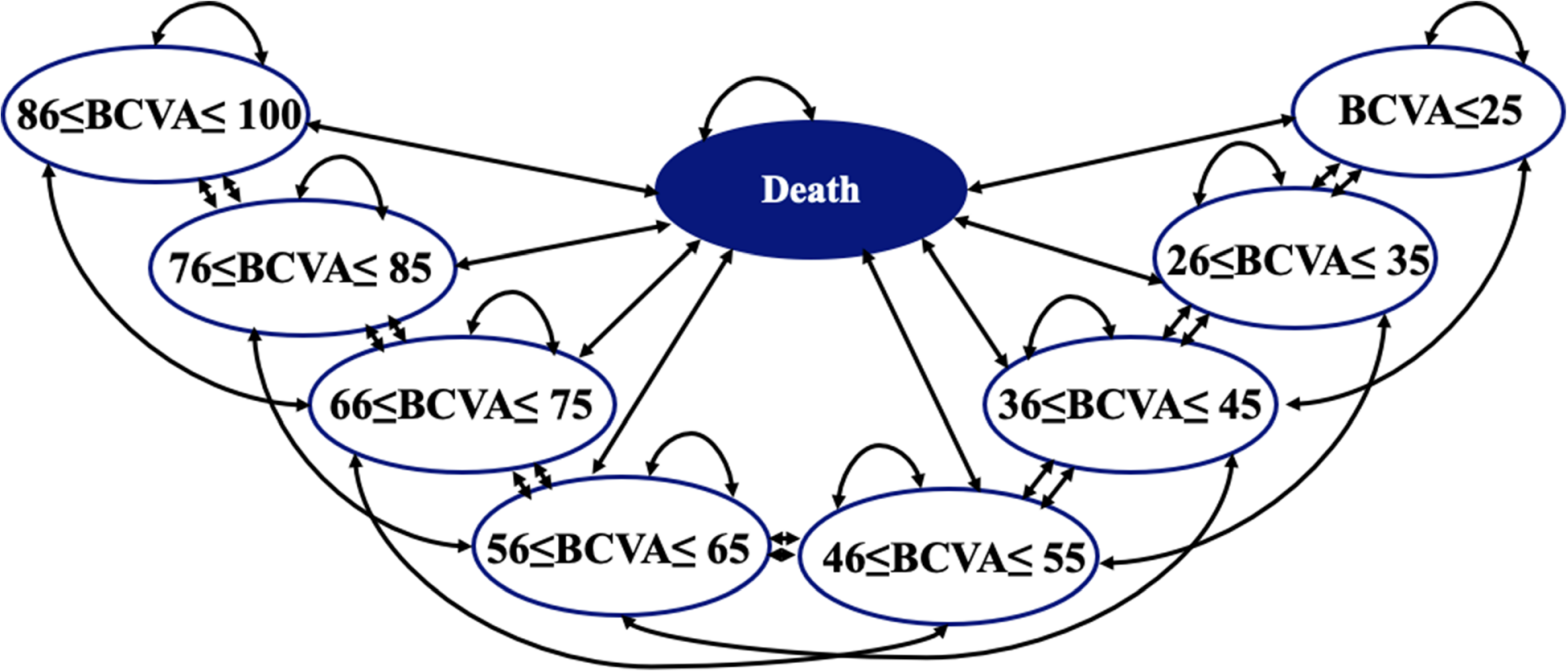
Model Structure. Early Treatment od Diabetic Retinopathy Study letter scale was applied in the model

A Markov model was adapted to simulate both Chinese BRVO and CRVO patient population using Microsoft Excel from the societal perspective. The model was reported in a previous study [9], containing eight BCVA health states defined using the Early Treatment Diabetic Retinopathy Study (ETDRS) letter scale and an absorbing state of death. Within the monthly cycle, each individual can move among different status (Fig. 1).

This model simulated changes in BCVA, quality-adjusted of life years (QALY), medical costs and direct non-medical costs of individuals with visual impairment due to BRVO or CRVO in lifetime. The starting age of BRVO population was 57 years and the starting age of CRVO patients was 54 year, which were the mean age years of patient population of corresponding pivotal clinical trials in China. The base-case analysis used an annual discount rate of 5% for costs and benefits following the China Guidelines for Pharmacoeconomic Evaluations. All costs were inflated to 2019 Chinese Yuan.

### Clinical input

The model’s clinical inputs in year 1 were obtained from the BLOSSOM and CAMILLIA trials (Table 1). Monthly transition probabilities were observed and calculated for each treatment arm, respectively. For the transition probabilities of the ranibizumab treatment arm, data from the 0.5 mg arm in BLOSSOM and CAMILLIA up to 12 months were observed. Similarly, transition probabilities for laser photocoagulation among BRVO patients were calculated using data from the control group of BLOSSOM trial for the first 12 months and the relative risk of laser photocoagulation to the observational-only care from a 2015 study [10]; thereafter, patients using laser photocoagulation treatment were assumed to follow the same transition probabilities as ranibizumab. For the observation-only group in CRVO model setting, transition probabilities were estimated using the control arm of CAMELLIA for the first 12 months. Assuming patients achieved stability at the end of year 2 except for the decrease in BCVA score because of the natural aging effect, ranibizumab effectiveness data in year 2 were taken as the transition probability at Month 12. Deterioration in BCVA since year 3 was from a population based observational study [11].

**Table 1:** Model input for effectiveness data

	BRVO		CRVO	
	Ranibizumab	Laser Photocoagulation	Ranibizumab	Observation
Month 1	BLOSSOM	BLOSSOM	CAMELLIA	CAMELLIA
Month 2–6	BLOSSOM	BLOSSOM	CAMELLIA	CAMELLIA
Months 7–12	BLOSSOM	BLOSSOM	CAMELLIA	CAMELLIA
Year 2	Horizon	Horizon	Horizon	Horizon
Year 3	Natural History	Natural History	Natural History	Natural History

The economic model tracked patients’ health status based on their BCVA scores in the treated eye, which could be either the better-seeing eye (BSE) or the worse-seeing eye (WSE). The starting distribution of BCVA levels were obtained from two BLOSSOM and CAMELLIA trials in China (Additional file 1: Table 1. More, according to the proportions of patients in the trials, the model assumed that 10% of patients whose BSE was treated at the beginning and the percentage raised to 21.5% at 12 months. The tariff scores used in the calculation of QALYs also varied depending on whether the BSE or the WSE was being treated. The model applied a weighted approach based on the number of patients whose BSE or WSE were treated.

### Utilities

Health related quality-of-life of patients with visual impairment is primarily associated with the BCVA score of their BSE. Utility scores of patients whose affected eye is their BSE eye are normally lower in patients whose affected eye is their WSE. However, utility gains from improving BCVA are generally considered to be higher among patients treated in their BSE eye than in those treated in the WSE. Thus, it was important to apply the corresponding utilities that were specific to BSE or WSE, respectively [12]. Utilities from BSE associated with BCVA letter scores were determined based on the algorithm developed by Czoski-Murray et al. [13] and were used as the base-case in the current model. In the absence of utility data for the WSE, patients were assumed to experience a maximum gain in utility of 0.30 between the best and worst possible health states [9] (Table 2).

**Table 2:** Utility Scores by BCVA Level

BCVA level	BSE utility	WSE utility
86–100	0.85	0.85
76–85	0.76	0.84
66–75	0.69	0.82
56–65	0.61	0.81
46–55	0.54	0.79
36–45	0.46	0.78
26–35	0.39	0.75
< 25	0.35	0.75

### Mortality

All-cause mortality from the life table for China was applied [14]. The relationship between BCVA scores and the relative risk to all-cause mortality had been demonstrated in previous studies [11, 15, 16]. When the BSE was the affected eye, the risk ratio (RR) was applied to the BCVA levels including less than or equal 35 letters, 36 – 55 letters and more than 55 letters. The RRs were 1.54, 1,23 and 1.00, respectively. When the WSE was affected, a RR of 1.23 was applied to all BCVA levels that were less than or equal to 35 letters and a RR of 1.00 was applied to the other BCVA levels.

### Resource use

Data on the frequency of ranibizumab treatment were collected from the BLOSSOM and CAMELLIA trails. Physicians from the 6 hospitals were interviewed on the treatment pattern of laser photocoagulation and observation-only care, follow-up visits and other medical services for RVO up to 5 years after the first admission (Table 3). The follow-up visits for BRVO patients could last for 5 years, while the follow-up visits for CRVO lasted for 3 years with a decreasing pattern.

**Table 3:** Frequency of healthcare resource use

	BRVO		CRVO	
	Ranibizumab	Laser Photocoagulation	Ranibizumab	Observation
Treatment visits				
First Year	7.0	1.4	8.2	0
Second Year	2.1	0.3	3.5	0
Follow-up visits				
First Year	2.3	4.9	2	5.4
Second Year	0.8	1.8	1.4	3
Third Year	1.1	1	0.8	1.3
Fourth Year	0.6	0.8	0.3	0.8
Fifth Year	0.2	0.2	0.3	0.3

### Costs

Unit costs of direct medical services were also obtained from the same survey, including the costs of ranibizumab, laser photocoagulation treatment, observation-only care, and follow-up visits (Table 4). A study in China demonstrated that the long-term annual non-medical costs and indirect medical costs were associated with BCVA levels [17]. When the BCVA score of the BSE was less than or equal to 35, the annual costs were ¥47,396 and when the score were between 36 and 55 letters, the costs were ¥30,849. With BCVA letters greater than 55, no additional non-medical or indirect medical costs were applied.

**Table 4:** Costs of healthcare services

Item	Unit costs
Drug	
Ranibizumab (unit costs)	¥3,950.00
Medical treatment services	
Laser	¥605.00
Administration of ranibizumab	¥776.00
Follow-up visit	¥664.00
Annual indirect medical costs and non-medical service	
BCVA 36–55	¥30,849.00
BCVA < = 35	¥47,396.00

### Productivity loss

Poor visuality caused by RVO may possibly lead to productivity loss of patients and requires additional care from family members or caregivers. As the present analysis was conducted from the societal perspective, the productivity loss of patients or caregivers were also taken into account. Similar to the resource use and costs data, the days of productivity loss on Chinese BRVO and CRVO patient population were also obtained through a survey at hospitals. According to the interviewed physicians, the productivity loss varied by BCVA levels. For BRVO patients in the health states of 26 BCVA letters and better, the average productivity loss from caregivers was 9.5 days per year. The corresponding number was 11.75 days for health states worse than 26 BCVA letters. The average productivity loss for BRVO patients associated with the two health states was 13.25 and 365.25 days per year, respectively. The average productivity loss from caregivers for CRVO patients were 13.25 days per year for all levels of BCVA. The productivity loss was 13.25 days per year for CRVO patients themselves when their BCVA were greater than 26 letters and better and were 365.25 when less than 26 letters. The total costs of productivity loss were calculated as the days of productivity loss multiplying the productivity per day per capita. Base on the GDP per capita in China 2019, the productivity per capita per day were ¥194.

### Sensitivity analysis

One-way deterministic sensitivity analyses were performed to evaluate the robustness of the model in the presence of uncertainty of key parameter values. Additionally, a probabilistic sensitivity analysis (PSA) was also conducted, the distributions of key input parameters in which are listed in Table 5. Beta-distributions were used for transition probabilities while gamma distributions were used for costs. Normal distributions were used for age and utility. The log-normal distributions were used for risk ratios. Each model was simulated for 5,000 times to generate an acceptance curve.

**Table 5:** Parameter values for probabilistic sensitivity analysis

	Mean	Variation	Distribution	α	β
General					
Starting age	54	5	Normal	n/a	n/a
% BSE at baseline	0.1	n/a	Beta	52.2	469.8
Quality of life					
Tx effectiveness probs—month 1	1	0.1	Lognormal	n/a	n/a
Tx effectiveness probs—month 2 to 6	1	0.1	Lognormal	n/a	n/a
Tx effectiveness probs—month 7 to 12	1	0.1	Lognormal	n/a	n/a
Comp effectiveness probs—month 1	1	0.1	Lognormal	n/a	n/a
Comp effectiveness probs—month 2 to 6	1	0.1	Lognormal	n/a	n/a
Comp effectiveness probs—month 7 to 12	1	0.1	Lognormal	n/a	n/a
Natural deterioration	0.00031	n/a	Beta	3.1	9996.9
Quality of life					
Utilities (all)	1	0.05	Normal	n/a	n/a
Costs					
Administration costs (all other treatments)	1	0.2	Gamma	25	0.04
Follow up costs (all treatments)	1	0.2	Gamma	25	0.04
Treatment visits year 1 (ranibizumab) BRVO	7.0	0.7	Gamma	100	0.07
Treatment visits year 2 (ranibizumab) BRVO	2.1	0.21	Gamma	100	0.021
Treatment visits year 1 (laser)	1.4	0.14	Gamma	100	0.014
Treatment visits year 2 (laser)	0.3	0.03	Gamma	100	0.003
Treatment visits year 1 (ranibizumab) CRVO	8.2	0.82	Gamma	100	0.082
Treatment visits year 2 (ranibizumab) CRVO	3.5	0.35	Gamma	100	0.035

The study protocol is performed in accordance with the relevant guidelines.

## Results

Treating BRVO with ranibizumab was shown to produce an increase of 0.646 QALY with incremental costs of ¥42,027 compared to laser photocoagulation over a lifetime horizon from the societal perspective. The incremental cost-effectiveness ratio (ICER) was ¥65,008/QALY, which was slightly lower than once the 2019 gross domestic product (GDP) per capita (highly cost-effective threshold, ¥71,000) and far below the three times the 2019 GDP per capita (cost-effective threshold, ¥213,000) in China. The CRVO patients using ranibizumab had a 0.551 QALY increase with an incremental cost of ¥36,244, resulting in an ICER of ¥65,815/QALY. Similarly to BRVO, the ICER was slightly lower than highly cost-effective threshold and far below the cost-effective threshold (Table 6).

**Table 6:** Base-case cost-effectiveness results

	BRVO			CRVO		
	Ranibizumab	Laser Photocoagulation	Incremental	Ranibizumab	Observation	Incremental
Total costs	¥87,924	45,952	¥41,971	¥139,407	¥103,163	¥36,244
QALYs	8.954	8.309	0.646	9.480	8.929	0.551
Incremental cost per QALY			¥65,008			¥65,815

**Fig. 2:**
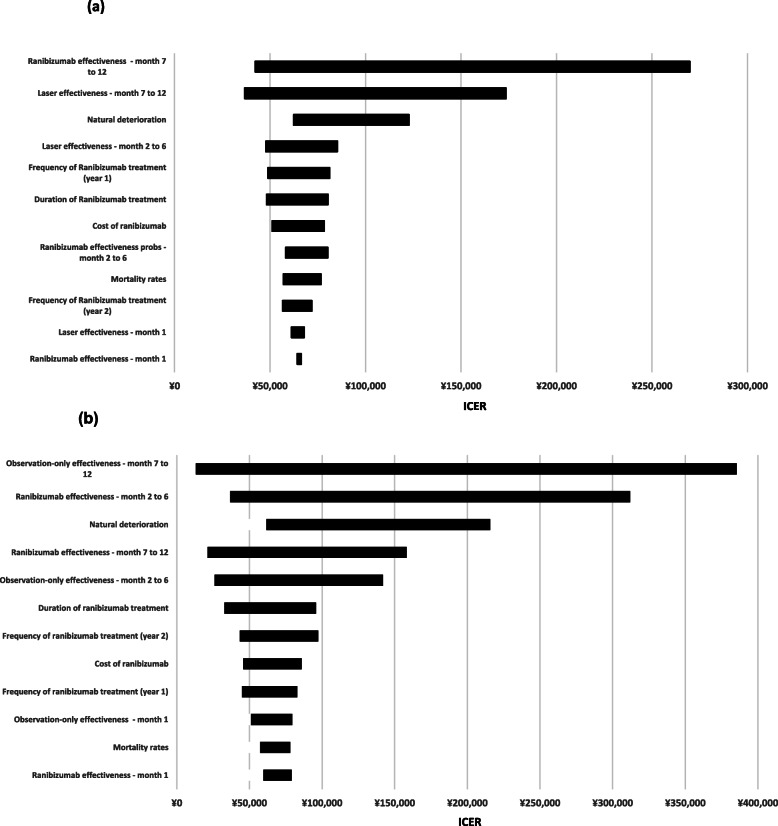
Deterministic sensitivity analysis tornado diagrams for **a** BRVO and **b** CRVO

The results of the deterministic sensitivity analyses are displayed in Fig. 2. As illustrated, the BRVO model is most sensitive to the treatment effectiveness of ranibizumab between 7 and 12 months in the first year. When it was set to 0.7-fold of the base-case value, the ICER reached ¥227,606 per QALY, which is higher than the cost-effective threshold (¥213,000). The sensitivity analysis of CRVO model are most sensitive to the 7–12 month effectiveness probability of the observation-only care and the treatment effectiveness of ranibizumab between 2 and 6 months. The ICER could reach over ¥213,000 when changing the value of the two parameters, indicating that ranibizumab could possibly be less cost-effective than observation-only care. The deterministic sensitivity analysis also demonstrated the ICERs could be affected through frequency of treatment, costs of treatment, and effectiveness probabilities in other treatment period besides 7–12 month. However, no changes in these parameters led to an ICER higher than ¥213,000.

**Fig. 3:**
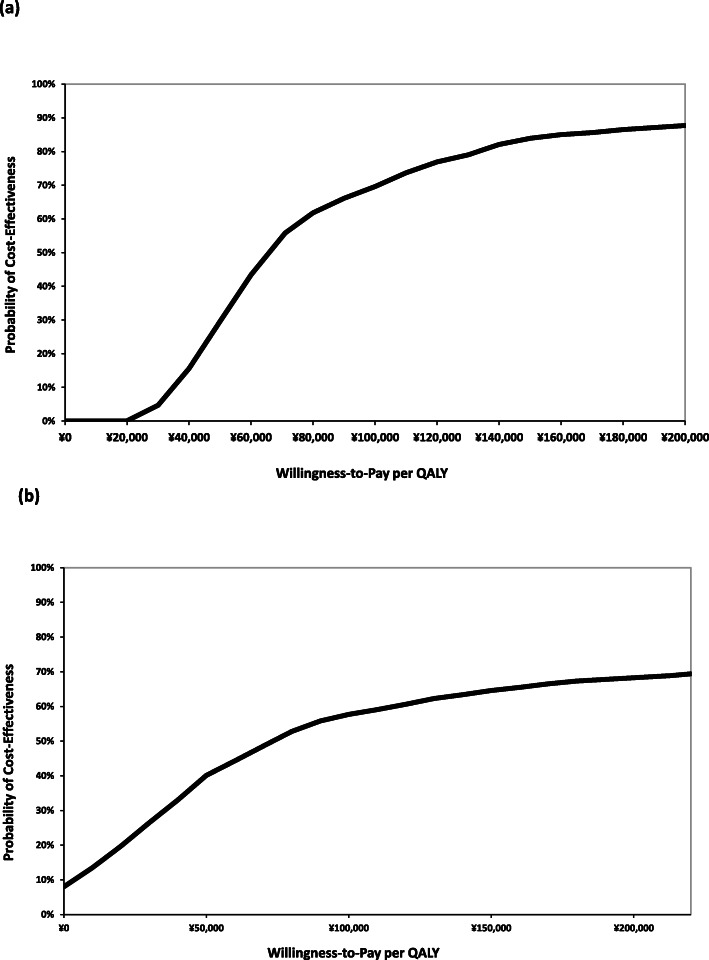
Cost-effectiveness acceptability curves for **a** BRVO and **b** CRVO

The acceptance curve of the BRVO model showed that the probability of ranibizumab being cost-effective is 55.8% with the willingness-to-pay per QALY at ¥71,000. After adjusting the willingness-to-pay per QALY at ¥213,000, the probability to favor ranibizumab increased to 86.5% (Fig. 3a). Meanwhile, the probability of ranibizumab to treat CRVO being cost-effective was 49.0% and 68.9% when the willingness-to-pay per QALY was ¥71,000 and ¥213,000, respectively (Fig. 3b).

## Discussion

The effectiveness inputs of the simulation model were mainly based on data derived from the BLOSSOM, CAMILLIA, and HORIZON (Cohort 2) clinical trials. The first two were designed and conducted for Chinese patient population. Thus, the data allowed a robust comparison of ranibizumab versus laser photocoagulation or observation-only care among RVO patients in China. The results demonstrated that ranibizumab was more cost-effective than laser photocoagulation for BRVO patients and confirmed that ranibizumab is also a cost-effective treatment for CRVO patients comparing to observation-only care.

A key strength of the current study is the healthcare costs and resource utilization were specifically collected among Chinese patient population. The economic impact of ranibizumab on RVO has been conducted among the UK population [9], while the results cannot be simply applied as a reference for Chinese population because the healthcare care delivery system, unit cost of health services, and labor productivity are significantly different between the two regions. Thus, Chinese-specific inputs are essential for an accurate estimation of the cost-effectiveness profiles of alternative therapies. Because of the lack of the data in literature, we interviewed ophthalmologists from hospitals in 6 large cities across China to represent healthcare costs and resource utilization.

Another advantage is the current model covers not only the impact on healthcare costs but also the societal productivity loss. As RVO is highly possibly cause blindness, it may consequently lead to heavy societal burden through productivity loss. Similar as local healthcare costs, we collected productivity loss related to RVO through completing physician surveys, as no such data available from previous studies.

The current analysis is subject to several limitations. First, the input values of utility across BCVA levels were based on international studies rather than Chinese-population specified investigations. Health related utilities normally vary across populations. Hence, international utility values may possibly lead to biased results. To overcome this potential disadvantage, the impact of utility variation was tested through sensitivity analysis. Second, although the effectiveness data of ranibizumab on Chinese BRVO patients were extracted from the BLOSSOM trial, the comparable effectiveness of laser photocoagulation were not directly available in the same trial. Thus, if the effectiveness is available in future clinical trials, the model needs to be updated. Finally, the cost-effectiveness of the current model is sensitive to the choice of willingness-to-pay thresholds. Although 3 times the GDP per capita is commonly used as the threshold of WTP, once the GDP per capita is also widely accepted as the highly cost-effectiveness threshold.

## Conclusion

The current Markov model demonstrated that ranibizumab is more likely cost-effective comparing with laser photocoagulation to treat BRVO and more likely cost-effective comparing to observation-only care to treat CRVO from the societal perspective when the three times 2019 GDP per capita in China are applied as the willingness-to-pay per QALY.

## Supplementary Information

**Additional file 1: Table 1**. Distribution of baseline BCVA levels

## Data Availability

The datasets analyzed in the current study are available from the corresponding author for reasonable requests.

## Abbreviations

BCVA: Best-corrected visual acuity
BRVO: Branch retinal vein occlusion
BSE: Better-seeing eye
CRVO: Central retinal vein occlusion
ETDRS: Early treatment diabetic retinopathy study
GDP: Gross domestic product
ICER: Incremental cost-effectiveness ratio
ME: Macular edema
PSA: Probabilistic sensitivity analysis
QALY: Quality-adjusted of life years
VEGF: Vascular endothelial growth factor
WSE: Worse-seeing eye
