# Supplementary material for: Cost-effectiveness analysis of ranibizumab for retinal vein occlusion patients in China from the societal perspective
Source: BMC Ophthalmol. 2021 May 24;21:229. doi: 10.1186/s12886-021-01997-1 (PMC8142632; doi:10.1186/s12886-021-01997-1)
Supplement: Supplementary file 1 — Additional file 1: Table 1. Distribution of baseline BCVA levels [file 12886_2021_1997_MOESM1_ESM.docx]

Additional file 1: Table 1. Distribution of baseline BCVA levels

| BCVA Level | 86-100 | 76-85 | 66-75 | 56-65 | 46-55 | 36-45 | 26-35 | 0-25 |
| --- | --- | --- | --- | --- | --- | --- | --- | --- |
| BRVO Patients | 0.0% | 0.0% | 25.3% | 36.6% | 21.5% | 12.9% | 3.2% | 0.5% |
| CRVO Patients | 0.0% | 0.5% | 13.8% | 33.8% | 27.0% | 14.3% | 8.5% | 2.1% |
